# Patriarchy and gender-inequitable attitudes as drivers of intimate partner violence against women in the central region of Ghana

**DOI:** 10.1186/s12889-020-08825-z

**Published:** 2020-05-13

**Authors:** Yandisa Sikweyiya, Adolphina Addoley Addo-Lartey, Deda Ogum Alangea, Phyllis Dako-Gyeke, Esnat D. Chirwa, Dorcas Coker-Appiah, Richard M. K. Adanu, Rachel Jewkes

**Affiliations:** 1 Gender and Health Research Unit, South African Medical Research Council, Pretoria, South Africa; 2 School of Public Health, University of the Witwatersrand, Johannesburg, South Africa; 3 Department of Epidemiology and Disease Control, School of Public Health, University of Ghana, Accra, Ghana; 4 Department of Population, Family and Reproductive Health, School of Public Health, University of Ghana, Accra, Ghana; 5 Department of Social and Behavioural Sciences, School of Public Health, University of Ghana, Accra, Ghana; 6 Gender Studies and Human Rights Documentation Centre, Accra, Ghana

**Keywords:** Intimate partner violence, Patriarchy, Gender-inequitable attitudes, Masculinities, Ghana

## Abstract

**Background:**

In order to reduce women’s exposure to violence and develop culturally appropriate interventions, it is important to gain an understanding of how men who use violence rationalize it. The present study sought to explore the perspectives of men who had used violence on their female partners, specifically their views on intimate partner violence (IPV), gender norms, manhood, their gender attitudes and to understand how these may drive male perpetrated IPV against women in the Central Region of Ghana.

**Methods:**

This was a qualitative study involving purposively sampled adult men who had participated in a household-based survey in selected districts in the Central Region of Ghana and who had self-reported perpetration of IPV in the past 12 months. In-depth interviews were conducted with 17 men.

**Results:**

Data revealed how a range of social, cultural, and religious factors – stemming from patriarchy – combined to inform the construction of a traditional masculinity. These factors included the notion that decision-making in the home is a man’s prerogative, there should be rigid and distinct gender roles, men’s perceptions of owning female partners and having the right to have sex with them whenever they desire, and the notion that wife beating is legitimate discipline. Findings suggest that it was through performing, or aspiring to achieve, this form of masculinity that men used varying forms of violence against their female partners. Moreover, data show that the men’s use of violence was a tactic for controlling women and emphasizing their authority and power over them.

**Conclusions:**

Developers of interventions to prevent IPV need to recognize that there is a coherent configuration of aspirations, social norms and behaviours that is drawn on by some men to justify their use of IPV. Understanding the perspectives of men who have perpetrated IPV against women and their motivations for perpetration is essential for interventions to prevent IPV. This is discussed as drawing authority from ‘tradition’ and so engaging traditional and religious leaders, as well as men and women throughout the community, in activities to challenge this is likely to be particularly fruitful.

## Background

In Africa intimate partner violence (IPV) is widespread [1]. Whilst there has been relatively little research on IPV conducted in Ghana, published studies have shown that more than one third of women report being abused in some form or another [2, 3]. A nationwide study on violence against women (VAW) in Ghana reported that 33% of women experienced physical violence at the hands of their current or previous partners, 29% of women had their first experience of sexual intercourse by force, whilst 33% of the women had been touched inappropriately against their will [3].

Women’s vulnerability to experiencing IPV is exacerbated by their relative lack of material resources, which creates dependency on male partners, as well as community norms of male dominance and acceptance of violence, with cultural ideologies that place women in subordinate positions [2, 4, 5]. These structures of domination and exploitation of women, which heighten their vulnerability to violence, obtain legitimacy from patriarchy [4, 6, 7].

In Ghana, the reports of exposure of so many women to violence points to a socially normative element in the use of IPV, yet its legitimacy is still contested. A recent study found that wife beating is commonly reported to be unacceptable, however a proportion of those interviewed asserted that it was justified in some circumstances, including when a woman disobeys her husband, neglects the children or refuses to have sex with her husband [8].

In the context of very high prevalence, these contested narratives of the acceptability of violence indicate both the need for interventions to prevent IPV and the possibility of building upon existing social disagreement to develop and deploy interventions working the discursive space of violence acceptability and the practical space of demonstrated assistance to women experiencing IPV. Understanding how narratives of acceptability of violence operate to create vulnerability for women and to justify the use of violence against them by male partners is critical for efforts directed at social change. This requires a much deeper understanding of how men who use violence understand and justify their behaviours, and the paths through which they seek legitimacy for it.

A starting point for research to understand men’s use of IPV is a theoretical understanding of Ghanaian society as deeply patriarchal [9]. There are distinct gender roles, with women expected to marry, bear children, keep the home and nurture children, whilst being available sexually for their husbands [8], while men are chiefly expected to work, earn and provide for their families [9]. Ghanaian patriarchy provides the framing of gender inequality and concomitant unequal power, social values, entitlements, and roles. In this context men construct and perform masculinities, which express their identities, aspirations and values, in their social relationships with other men as well as with women. Connell [10] argues that in any setting there are multiple masculinities, all of which draw on the patriarchal privilege, but they have an uneven relationship to domination and control over women, and also are held with unequal esteem by other men. Some masculinities are much less violent than others, and emphasise to a greater extent men’s responsibilities and position them in a supportive and cooperative relationship to women. Others are predicated much more strongly on dominance and control over women, often with men’s success and honour viewed through the lens of their ability to do this, and violence or threats are strategically used to achieve this position over women, teach them gender hierarchy and punish transgressions. These, and other, different models of manhood sit uncomfortably side by side within a community, with their relations unequal and contested. Among them there is usually a communal cultural model of masculinity that is most widely recognized as an ideal and superior to the other masculinities [11, 12]. This is described by Connell as hegemonic masculinity [10]. In the hierarchy of masculinities, the dominance maintained by the hegemonic masculinity is attained through a social agreement, rather than through violent suppression of the subordinate masculinities [13, 14].

Hegemonic masculinities, as an ideal, reflect a masculine position which is aspirational as often as it is occupied. For example, in most societies it includes elements of ideas of men as providers, and these are esteemed and aspired to by both men who are financially able to occupy this role as well as by men who may be marginally- or un-employed and struggle to do so. In sub-Saharan Africa hegemonic masculinities invariably express men’s power and dominance over women, but the role of men’s violence in achieving this is contested. Wood et al. [15] argued that amongst the Xhosa in South Africa it is men’s power of verbal persuasion which is more highly regarded, although violence may be used at times when persuasion fails. In other settings or among other groups within that setting, violence may be more readily deployed by men. There is considerable debate in the literature [16] about whether the use of violence makes men feel good, and much as it is often legitimated as ‘discipline’, it is often asserted that it does not if viewed as ‘loss of control’. A further pertinent element of this debate is elucidation and understanding of how men justify their use of violence to deflect blame for it, where they see the act as blameworthy, and to deflect the stigma that might otherwise be accorded to men who ‘lose control and are violent’ [16].

For those who seek to end women’s exposure to IPV, and develop culturally appropriate interventions, it is critical to gain and understand the perspectives of men who perpetrate IPV against women, their motives and justification for perpetrating it. In this paper, we draw on the narratives of men who acknowledged their use of violence over their wives and girlfriends in Ghana and show how a range of social, cultural, and religious factors – flowing from patriarchy – intersected to inform the construction of a traditional masculinity, and how men draw on this to legitimize their violence.

## Methods

### Study design

This qualitative study was conducted during the pre-intervention phase within a larger trial that assessed the community level impact of the Rural Response System (RRS) intervention (registered on ClinicalTrials.gov Identifier: NCT03237585). The baseline survey for the larger trial used a multistage stratified cluster random sampling process to select participants. Full details regarding the sampling approach for the larger trial are described elsewhere [17, 18].

The primary objective of the RRS is to reduce and prevent VAW in Ghana. The RRS uses the strategy of trained community members known as community-based action teams to undertake awareness-raising on VAW as well as providing support to victims of violence to access justice. For this study, in-depth interviews (IDI) were conducted with men who had self-reported in the quantitative baseline survey to have perpetrated IPV (*n* = 17 IDIs).

The scope of inquiry for the IDIs (see appendix 1) was to understand the perspectives of the men in our sample – with regards to IPV, their views on gender norms, manhood, and their gender related attitudes and practices, and whether and how these drive male perpetrated IPV. The questions which were asked in the interviews were developed by the authors of this paper for the purpose of this study.

### Setting

The trial was conducted in four districts located in the Central Region of Ghana. Two of these districts are along the coast while the other two districts are inland districts. The four districts in the study (each comprising about 10 communities) were selected based on operational and program considerations. Selection of participating districts was done using a census map of the Central Region that showed Inland and Coastal districts.

After excluding some districts because previous intervention work on VAW has been carried out in those districts; two inland and two coastal districts were then purposively selected as study sites. Designated sites were separated from each other by a geographical buffer (at least one district wide) to reduce spill over*.* Adult literacy rate in the Central Region is about 50%, with more men being literate (69.8%) compared to women (46.3%) (Ghana Statistical Service, 2013). The region is predominantly Akan speaking (82.0%) and Fante is the indigenous dialect of most districts in the region. Unemployment rate is 8.0% which is 2.4% lower than the national average.

### Study participants

Research assistants were trained and primed to identify men who reported experience of IPV (as perpetrators) during the baseline survey. These respondents were followed-up and privately asked to confirm if they had perpetrated any form of IPV (physical, sexual, emotional, economic) in the past 12 months. Those who admitted to such behaviour were invited to participate in the IDIs, which would offer them an opportunity to share their experiences within a private space.

The same research assistants contacted those who agreed to this invitation to conduct the IDIs. Focusing on such individuals allowed us to capture specific IPV experiences in these communities and examine how these experiences highlight the knowledge and understanding of the negative impact of IPV as a social issue as well as actions taken. All IDIs with men were conducted within the intervention communities. While the research assistants came from similar communities as participants, they did not conduct interviews in their own communities and had no prior knowledge of the participants.

### Participants’ characteristics

Seventeen men undertook IDIs, majority of whom were married or cohabitating, professed Christian faith, and had attained junior high school level of education (grade 7–9). With regards to age, they ranged between 18 and 73. Each of the 17 men was interviewed once. Few of the participants had lived less than 5 years in the community, while most had lived there over 16 years. In terms of age, four men were between the ages of 18–25, six were between 26 and 35 years, three were between 36 and 44 years and six were between 45 and over. With regards to marital status, 13 were married, three were single, two were cohabiting and one was divorced. One had never attended school, six had completed primary school, three had completed junior high school, five had completed senior high school, two had tertiary education.

Amongst those employed, most men were likely to be working in the agriculture sector (including fishing) as it was the main occupation and employed more than two-thirds of the work force in many districts. Fishing is concentrated mainly along the coast, whereas cocoa and oil palm production is concentrated inland.

### Data collection

The IDIs were carried out from May to June 2016 by male interviewers. The interview guides were translated into local languages (Fante and Twi) by bi-lingual members of the project team at the University of Ghana. This was independently back translated by consultants who had not seen the English guides. The project team then discussed and resolved differences before the tools were used. Participants were interviewed in either Fante and Twi, in a private space of their compound or away from their residence (if desired). Interviews lasted about 1.5 h. From about the 15th interview, no new information was being produced, and this suggested to us that saturation had been reached.

### Data analysis

All audio-recorded interviews were transcribed verbatim. Transcriptions were augmented with researcher’s field notes generated through observations during the IDIs. Data were analysed inductively using thematic analysis [19]. However, there were deductive elements to the analysis as we explored themes that have been reported in similar published studies and then tested those in our data.

Transcripts were read repeatedly, and initial codes developed based on the IDI guide and short words or phrases representing segments of the text in the transcript. The first author used the codes to develop a codebook. Following this stage, all authors reviewed and tested the applicability of the codebook using the raw data from the transcripts, which led to expansion of codes. Next, text which seemed to fit together was grouped together under a specific code [20].

Further to this, the authors explored the data and identified numerous open codes. Analogous open codes were grouped together under clearly defined categories [20]. Next, the authors explored the relationships between the categories and interpreted what they saw emerging [19].

### Ethical considerations

Ethical approval for the study was obtained from the Institutional Review Board at the Noguchi Memorial Institute for Medical Research, University of Ghana, (# 006/15–16) and the South African Medical Research Council’s Ethics Committee (EC031–9/2015). All project staff received training on gender, VAW, and research ethics prior to implementing the trial. All participants were asked to provide written informed consent before participating. This was done in participants’ language of preference (English or local languages).

Participants were informed about the purpose, risk and benefits of the study and that participation was voluntary. They were also informed that they may withdraw at any stage or skip any question in the research, with no adverse consequences to them. All participants were assured that the information they provide will be handled confidentially and that findings will be reported with complete anonymity (pseudonyms were used for participants during interviews and are not representative of any particular ethnic group).

All interviews were recorded both in writing and audio taping with the consent of the participants. Participants were reimbursed for their time with a beverage (malt) and water as well as Ghs 10 (~ 2.5 USD).

## Results

The sample of this study was limited to men who reported in the baseline survey to have used violence on their female intimate partners. As such, these findings reflect the gender attitudes and practices of men who reported to have used IPV, and not those of all men in the Central Region of Ghana.

### Men make decisions at home and perform his roles

Most men held a view that decision-making in the home is a preserve of men, and also a responsibility men cannot abdicate, as it is part of their expected gender roles. This was best illustrated in Dodzi’s narrative to follow: ‘*At home I make the decisions and perform my roles and do the things that I’m supposed to do for my wife when it comes to our chop money* [i.e. money for food and the home and other things]’. Several men viewed decision-making in the home as a man’s natural and essential role, as Adjo explained: ‘*In most cases, it’s the man who has to initiate such issues of planning for the future of the children. If that fails to happen, the future of the children will be jeopardized*’.

Our interviews suggested that a man’s failure to provide such direction to his family would reflect badly on him as a man, in so doing call into question his masculinity. Yet, on the other side, this socially sanctioned duty of men to provide direction to his family primarily worked to elevate and emphasize men’s position as superior to women and other males (e.g., boys) in the gender hierarchy. Elikplim’s asserted: ‘*The man can tell his son not to go out and he’ll listen but the woman can also tell the son the same thing but the son won’t listen to his mom*’.

Men considered that the hierarchical gender structure described here, with men occupying the top most position, needed to be respected by the wives to prevent violence in their homes. Any challenge to it from a wife could result in beating from her husband. To argue and substantiate their view that a man is the head of the household, some men drew on religious teachings and texts. Danquah contended that: ‘*As Christ is the head of the church so is a man to the house*’. Several of the men drew on the higher authority of ‘God’s law’ to justify their view that they were superior to women. Adjo commented that: ‘*If we are to be honest, even the Bible makes us understand that the man is the head of the family …* ’. For these men, this ‘God given’ role could not be contested, and this meant that women must subject themselves to their control, and dutifully perform their duties to serve them. It was in the context of a perceived challenge to men’s rules and authority that men emotionally and or physically harmed their female partners.

### ‘Women are not regarded in society’

While Acheampong explained that in his community ‘*men do not treat women as fools’*, and ‘*men are not rough with women*’, other men shared contrasting attitudes and experiences regarding gender relations between men and women in their communities. They shared narratives that suggested they thought women were sub-human or intellectually inferior. This is evidenced by Elikplim’s argument:

Elikplim: I think that since women are not regarded in the society that’s why they’re not allowed to make decisions; we count them as part of our cooking utensils and other things.

Interviewer: So, are you saying that women are counted as cooking utensils and other things?

Elikplim: Maybe this is one the woman 2, like plate 1, woman 2, and spoon 3.

Highlighting that women were feeble-minded and sub-human, men like Elikplim contended that it was the men’s duty to think on behalf of women and provide them with direction on what ought to be done and how. Yet, while some men reasoned that it was progressive for a man to consult with his wife when taking decisions, they stressed that the final decision-making lies with the man. Abedi underscored that: ‘*A man [takes the decision] but a woman also brings her idea to touch, so it’s going to be 70-30*’.

Similarly, other men argued that it is important for a man to listen to his wife’s opinions as that can aid men in making well thought through decisions. However, it was apparent in the data that the final decision on a matter rested with the man. While initially it may appear that these men were different from those, within this sample, who demonstrated gender-inequitable attitudes with regards to decision making at home, these men had no intention of considering the views of their wives and allowed no indication that their wives had the power to make them decide on anything that did not suit them. Also, interviews showed that the men’s trepidation about including their wives in decision-making process stemmed from how they felt the society would perceive them and appeared to have nothing to do with the quality of the woman’s contribution.

Our interviews suggested that almost all men interviewed preferred a wife who exhibited a femininity characterized by obedience towards her husband. This is best illustrated in Abronoma’s extract to follow:

Decisions normally taken are farm related. The man could take a decision like “I have seen some farm somewhere here, let’s go clear the land”. If the woman is “correct” [obedient] she will go and assist you cultivate the land. On the other hand, if she is not correct [disobedient], she will not help the man in the farm. (Abronoma)

The notion that a good woman acquiesces to men was a strategic tool in bolstering this traditional masculinity and worked to sustain these men’s dominance over women. Practically, the men’s stated preference for a subservient wife was another tactic for controlling women and ensuring that they be fearful and subservient to their husbands, and thereby conform to their dictates. The ever-present threat of violence underpinned this.

Interviews suggested that wives, however, were not completely excluded from decision-making. We interrogated the data to understand the terms and circumstances under which women were included. The narratives suggest that instances of a wife’s inclusion had conditions attached, and chiefly depended on the woman’s demeanor. For example, wives who were perceived to be disrespectful by some men, and disagreed with them were excluded, while those viewed as subservient were included. In his interview, Abedi explained that he broke his engagement with his fiancée when he noticed that she had started disrespecting him and challenging his decisions:

We were planning to get married but later I gave one of my keys to her but she felt pompous; if I said ‘oh Abena do this’, she said she won’t do that. She started challenging me so I told her I can’t continue this thing … she can go her way. So as for women, if you study her well and you get married to her, you can share ideas. That’s why most men don’t share ideas with their wives, only a few. (Abedi)

Thus, the women’s inclusion in decision-making was a reward for their subservience and support to men’s ideas, a subtle strategy to control women and keep the existing hierarchical gender structure undisturbed. It appears that the wives of these men knew of these expectations, were aware of the violent consequences to them if they challenged the husband’s authority.

### Men should provide, and women must cook

The interviews suggest there were rigid and distinct gender roles which were conspicuously reflected in the attitudes and practices of these men. Men’s dominance of decision-making was one male gender role, but there were many more that were perceived as ingrained in inflexible and unchanging ‘culture’. For example, Aboagye asserted that in his culture there were ‘*responsibilities that were solely reserved for women and others exclusively meant for men*’.

The gender roles for women centered around the home – and this is best shown in Adjo’s explanation that: ‘*The difference here is, in the case of a woman, your responsibilities are to clean the home, tidy up everywhere including the toilets, and cook for the man*’. In the interviews, it appeared that the need to reinforce men’s supremacy over women, primarily informed the gender roles for women. For these men, women’s responsibilities were menial and domestic.

On the other hand, there were roles that were clearly reserved for men. Many men expressed the view that a man’s place is outside the home; where he is expected to toil to earn money for his family’s upkeep. This provider role – a key trait of the traditional masculinity which was valued by most of the men comes with clearly stipulated responsibilities that a man must perform. Aboagye’s assertion that: ‘*The role of the man is that since you brought the woman into your house, you have to make sure you provide her with food, clothing, good shelter, and other basic things at home*’, is explanatory.

As argued by Adjo below, a man who succeed in performing these responsibilities obtained some social rewards including respect and honour as a man in his community:

In terms of reward, if there is any that comes, it goes a lot more to the man than the woman because your ability as a man to take very good care of your wife for her to look good is what brings honour to you. (Adjo)

Yet, interviews suggest that a man who failed to meet these responsibilities may lose the respect of his wife and therefore be stripped, symbolically, of his position as the head of the household.

Gender roles could, however, be temporarily filled by either men and women. Yet, per these men’s views, this exchange of gender roles chiefly depended on men’s will. Some men emphasised that such change was time and circumstance bound and never perpetual. In explicating this, Aboagye said:

It will work because it could happen that there is some day the woman wouldn’t be feeling that ok so since you the man knows she is sick and cannot do her household duties, you have to give a helping hand maybe for 2 or 3 days. In the same way when it comes to the woman, you have noticed that the man isn’t feeling well so the roles or duties he is supposed to do that period and he isn’t doing, you have to help him accomplish that task for maybe 2 or 3 days.

In line with Aboagye’s view, in other men’s narratives there was an indication that gendered tasks could be shared in a limited way under certain circumstances. For example, Kofi emphasised that a man should help with domestic chores when the wife was infirm and therefore unable to carry out her domestic roles but not in other circumstances. He posited:

The role of the woman is that you have to cook, you can also fetch water for your husband to take his bath and ask him to eat his food. You the woman has no right to ask your man to go to the kitchen and cook food. What is even the essence of the marriage in the first place? The only time you can sacrifice and do those things is when you realise that your wife is sick and cannot do anything. (Kofi)

In the interviews, it was evident, however, that the rigidness of the gender roles was challenged under various economic conditions especially when the woman was bringing in money in the home or was the sole breadwinner, but even here it was implied that this could be temporary. Aboagye’s extract below shows this:

Sometimes when you the man is financially unstable, you can at least talk to your wife to support with what she has, and later you will pay her back. So, I think when all these go on in the house there will be peace. (Aboagye)

Linked to the notion that the men’s place is outside the home, material provision was a critical feature of the traditional masculinity, valorized by most men in our sample. To attain this masculinity, men had to provide for their wives and children. Any failure to provide triggered frustration in their wife and, sometimes, a violent response from the men. Kofi’s extract to follow is illustrative:

Chop money can also generate violence in the house. Maybe even if the man is financially unstable, he [husband] won’t even inform the woman of the fact that he doesn’t have money today but the moment the woman asks for money, then he will use pride. Pride can never solve that problem. (Kofi)

If the wife questioned the men’s inability to provide for her and his children, men interpreted this as a direct assault on his masculinity and would use violence to temporarily reclaim his honour.

### She is mine and must allow me to do it (sex)

In the interviews, several men emphasized that the wife’s responsibility towards her husband centered around her ‘pampering’ him. According to these men, ‘pampering’ meant to sexually pleasure and satisfy the male partner. Two reasons appeared to inform these men’s sexual entitlement on their female partners: the bride-price and the notion that ‘men own their female partner (i.e. ‘You are my girl’)’. In the interviews, men interpreted their bride-price payment as meaning that they own their wives, and that their wives cannot refuse their sexual advances.

Our data showed that men’s entitlement to be ‘pampered’ by their wives was inextricably tied to their view that because the man had paid bride-price for his wife and met other customary demands from the wife’s family, he was entitled to her sexually. For men who held this notion, a wife was thus culturally bound to satisfy her husband sexually whenever he desired. Adom explained: ‘*How can I marry you and put a ring on your finger and when I want to have sex you say no? If you won’t do it then it means you don’t love me*’.

The payment of bride-price emphasized men’s sense of ownership of their wives and entitlement to their bodies. It also meant that wives were not permitted to go with other men. For men like Danquah, a woman who was suspected, or known, to have cheated on her husband should expect to be physically beaten. Danquah posited:

The woman I was with cheated on me with another man as a result I got annoyed and gave her some punches. Because, as a woman that I have married legally, how can you cheat on me? So, if a woman goes out without coming to sleep home, what does it mean? So, that is what happened which led to the violence. I gave her some punches which she also replied and become a problem which led to the divorce. (Danquah)

It was not only in a marriage that some men felt sexually entitled to their partners. Men in dating relationships also felt entitled to have sex with their partners whenever they desired sex. Several men reported that they had forced their girlfriends to have sex with them.

It appears their entitlement to have sex with their girlfriends, whenever they wished, was also linked to their view that men own the women they are in a relationship with. Such sexual entitlement and sense of ownership of women is best illustrated in Abedi’s extract below:

Oh I forced her I told her, ‘chaley [dude] today I feel oo’ so I want to have sex with her, but she said ‘chaley today I am tired’, but I said ‘oo what are you talking about, that is why I told you I am in love with you, you are my girl so you have to allow me to do it’, but as I said I had to force her … yeah, about 3 months, 4 months I hadn’t had sex so (laughing). I forced her. (Abedi)

In our sample, men who reported to have sexually violated their partners were likely to also report that they felt entitled to their partners’ body, that their partners were their possessions, and had strongly identified with the traditional masculinity circulating in this context.

### Beating as discipline

The men in our sample viewed partner violence as a strategy to discipline a female partner. Addae very simply remarked: ‘*occasionally the man will beat the woman’.* Danso similarly explained that: *‘in marriage it is not always smooth, there will definitely be beatings, but they are no big issues*’. Through first diminishing and even dehumanizing women, men found it easier to inflict violence on their female partners.

For some men, wife beating was an entitlement that came with marriage, and was closely tied to men’s payment of the bride price for the wife. Adom’s quote below shows this:

When I used to stay here the relationship that exists between a man and woman sometimes it’s heart-breaking … because a man can be living together with a woman while he has not performed the marriage rites, but the man beats the woman like he has performed the marriage rites of the woman. (Adom)

Wives were perceived to be men’s possessions and thus under their control. Dodzi explained that: ‘*for understanding to prevail in the home, she [wife] has to obey me and listen to what I tell her*’***.*** When a wife was judged to have misbehaved, or had been disobedient, men felt it was their responsibility to discipline her. Abronoma’s narrative to follow evidences this, he posited: ‘*I came from the farm only to find my wife sleeping instead of joining me in the farm. Meanwhile, she wasn’t sick … nothing happened. She wasn’t injured. I gave her a lesson [beating]*’.

Likewise, several other men described instances where they had beaten their wives. Abronoma reported that ‘*My second wife, I caned her … yes. I used a cane to whip her’.* Suggesting that he has beaten his wife multiple times, he further said: *‘ … the last time it [beating the wife] happened was when I divorced my last wife*’ (Abronoma). That some men had repeatedly beaten their wives and used implements (such as a whip) to do this with, suggests the severity of IPV perpetration by these men.

Men felt they had the responsibility to discipline a ‘*lazy wife*’, and some, like Mawuli, went as far as to blame the women for their violence: ‘*[it] was because of what she said that made me raise my hands on her*’. He further explicated: ‘*Okay, I see some cases of these violent acts. Sometimes the woman is someone who doesn’t respect, so the man can hit her in the face or be physically violent towards her*’. He felt it was his responsibility to beat his wife when she ‘*deserved*’ it, and complaining about being hampered in doing this due to the children, Mawuli said:

I have hit her [wife] before.

Interviewer: Was it because of her refusal to sleep with you?

Not only that but other issues also gave rise to my violent behavior towards her … because of the kids at home I find it difficult to hit her even if she does wrong. (Mawuli)

Blame deflected any sense of guilt for the beating. When Kafui was asked how he felt after beating his partner, he stated that ‘*I didn’t feel anything because; I knew she wasn’t doing the right thing and if she had listened to me, I wouldn’t have beaten her*’. Beating their wives was a necessary form of discipline for a disobedience or shirking responsibilities, something that men could not abdicate. For them, it was part of being a man. Notwithstanding, few men perceived wife beating as violence and somewhat regretted it.

## Discussion

This study has described the perspectives of men who used violence against their female partners. We have shown how an array of social, cultural, and religious factors – deriving from patriarchy – coalesced to inform the construction of a traditional masculine position which the men interviewed aspired to occupy. These factors included the notion that decision-making in the home is a man’s prerogative, as are the rigid and distinct gender role (e.g. domestic work), men’s perceptions about owning their female partners and having the right to have sex with them whenever they desire, and the notion that wife beating is legitimate and important for discipline. Our findings show that it was through performing or aspiring to achieve this form of masculinity that men employed different forms of violence against their female partners. Moreover, findings suggest that the traditional masculinity displayed or aspired to by these men was rooted in patriarchal power relations in which men were positioned as superior to and dominant over women. Also, findings revealed that patriarchy was particularly reflected in the gender attitudes and practices of the men, who had reported that they have been violent towards their partners and had generally supported men’s dominance in decision-making at home and wife beating.

These findings are an important contribution to literature as they highlight how some structures of patriarchy (i.e. men’s violence and patriarchal mode of production) [21] play out in this setting to subordinate women and inform the construction of a traditional masculinity (amongst men in our sample) – which often manifested itself through display of extreme gender-inequitable attitudes and use of various forms of violence against female partners. Thus, these findings bring significant nuance to understandings of the links between patriarchy and male perpetrated IPV in the Central Region of Ghana [22]. Furthermore, these findings shed light on the ideas and justification violent men in this setting drew on to support their use of violence against their female partners and deflect blame.

It has been argued that Ghanaian culture demands that women should not only be submissive to their husbands, but also demonstrate unquestioning respect, be dutiful, and serviceable to the extent that going against or challenging abuse may be interpreted as an attempt to disrupt the authority of the man [7, 9]. In this study, what the participants described is an inflexible gender hierarchy, which is enforced, as we have shown, through authoritarian rule by some men in the home.

In this context, the use of violence against female partners is seen as an integral part of the dominant masculinity, as presented by these men who have all themselves used violence. Hearn and others have argued that the use of violence by men is not an inherent part of hegemonic masculinity [23, 24], but our data suggest that in these contexts in Ghana it was inseparable from an authoritarian patriarchy and an intrinsic element of the expected and revered authoritarian rule by some men within the home.

Our study has shown that the structures of patriarchy (e.g. men’s violence and patriarchal mode of production) and related elements and practices illuminated by the interviews were very similar to those described by Walby [21] and found in very many settings: distinct gender roles, decision-making being a men’s prerogative, men’s sexual entitlement, and wife beating as discipline (see also [25]). However, these elements alone, reveal little about the specific cultural context in which men construct and perform their masculinity and the embodied meaning of these different elements.

Notable features from the interviews were the extremes of subordination of women – some of the men dehumanized their female partners or viewed them as feeble-minded, categorizing them as possessions, little more than animated household implements. This clearly constitutes pervasive emotional violence against women. Some of the authors of this paper have undertaken research on IPV in many countries over several decades and have often reflected that women are objectified and treated as children or as possessions, but have not previously encountered women described as household implements in any setting. Furthermore, the subordination and dehumanization described by men interviewed – a tactic often used in genocides or conflict situations [26] – was required in order to justify the treatment of women, when the men thought women were disrespectful, disobeyed or had transgressed gender roles. These men said it was their culturally sanctioned responsibility to control and discipline their female partners, as part of fulfilling their gender roles [27]. Feminist scholars argue that when the sub-humanness of women obtains justification from tradition and religion, it legitimizes men’s use of violence against women; and that, when men think of women as less human, they often act in violent ways towards them (Shose Kessi: personal communication). Indeed, some men’s sexual violence against their wives appeared to have been legitimated by the cultural practice of bride-price payment which, in turn, seemed to have informed these men’s sense of ownership of their wives, making them to believe they had unlimited access to their wives’ bodies [28]. It was thus in the context where wives resisted their husband’s sexual advances that men sexually assaulted their wives [5, 7]. This is akin to findings of a South African study in which men’s sexual entitlement was the most commonly reported motivation for rape of women [29].

There are implications for the development of prevention interventions that stem from this research. To build effective programmes to stop men’s IPV in this setting, more research is needed to understand how men who are violent perceive dominant forms of masculinity and their contribution to IPV. It is difficult to see how interventions to prevent IPV can be effective without transforming the dominant construction of masculinity. For as long as men perceive that they are expected to enact authoritarian rule in the home, and view women as inferior to them emotional, sexual and physical violence are likely to be inseparable parts of the domestic regime. Building respect for and admiration of women, acknowledging domestic partnership and promoting shared decision-making are essential elements of programmes that seek to disrupt authoritarian patriarchies and prevent male perpetrated IPV. In this setting, such programmes should also address cultural and other context specific factors that drive male perpetrated IPV. Furthermore, among these violent men, who confidently perceived themselves to be ‘traditionally’ masculine men, widespread community-based change is most likely needed to enable and sustain change in masculinity, drawing on contributions from traditional and religious leaders as well as other community members.

We have also shown that some men refrained from beating their wives in front of the children or regretted beating their wives. This is an important finding as it displays a possible “crack” in the solid construction of violence as an intrinsic part of the hegemonic masculinity in this setting [14]. This suggests that the dominant masculinity circulating in this setting was fragile and open to challenge [14], and this needs to be emphasized as a factor that could potentially protect women from IPV. Moreover, this opens a window of opportunity for prevention interventions in those areas where it is difficult to tackle rigid gender norms upfront. Using violence within the family as an entry point should thus be an integral component of gender-transformative interventions to prevent male perpetrated IPV in Ghana.

### Reflexivity

The authors constitute a team of African women and men researchers, who are situated within both academia and practice on the continent. They come from diverse disciplines and have expertise in qualitative research. The authors who are Ghanaians were born in Ghana and had lived within this context for the most part of their lives as academics and researchers. They were thus not only familiar with the context but had also spent a considerable amount of time in the study communities during the data collection period. With appreciation that their previous observations may bias their viewpoints on IPV perpetration in Ghana, they were fully aware and worked hard to be impartial in the interpretation of the data and avoid unintentionally imposing their personal opinions and assumptions on the data. Moreover, all authors including those who were African foreign nationals were a part of this study from conceptualization and data gathering through to the analysis and write up of the paper.

### Limitations

In this study, we only interviewed men who had reported in the survey to have perpetrated IPV against a woman. This may explain their extreme perspectives on IPV, gender-attitudes, gender norms, and violent practices; and these men are likely to be different in terms of gender attitudes from other men who do not perpetrate IPV in the Central Region of Ghana. However, the data from the men’s interviews were triangulated with the researchers’ observation notes – which were documented during the fieldwork – and these notes were included as data in this analysis.

As this was a qualitative study, which interviewed men who had perpetrated IPV, our findings are not generalizable, however we hope the insights we have gathered are of interest to other settings. It is also possible that some men may have felt compelled to answer in certain ways, including feeling the need to describe themselves as macho or to represent themselves in a socially desirable manner with regards to their perspectives on gender relations with women and use of violence on their female partners. Notwithstanding, this study is notable for the unique sample it used to study qualitatively the drivers of male perpetrated IPV against women.

## Conclusions

There is consensus in the field of IPV that to reduce male perpetrated IPV, social norms that hinder gender equity and foster violence should be addressed [22, 30]. In Ghana, there is need for evidence-based primary prevention interventions that aim to disrupt and/or dismantle the existing hierarchical gender structure and the harmful hegemonic masculinity circulating in this setting. To reduce male perpetrated IPV in Ghana, there needs to be urgent implementation of evidence-based community-based interventions that aim to change destructive social and gender norms that render women subordinate to men and legitimate male violence, among which the COMBAT intervention that was evaluated as part of this research shows promise.

## Supplementary information

**Additional file 1.**

## Data Availability

The datasets used and/or analysed during the current study are available from the corresponding author on reasonable request.

## Abbreviations

VAW: Violence against women
IPV: Intimate partner violence
RRS: Rural Response System
RCT: Randomized controlled trial
IDI: In-depth interview
FGD: Focus group discussion
