# Supplementary material for: Patriarchy and gender-inequitable attitudes as drivers of intimate partner violence against women in the central region of Ghana
Source: BMC Public Health. 2020 May 13;20:682. doi: 10.1186/s12889-020-08825-z (PMC7222313; doi:10.1186/s12889-020-08825-z)
Supplement: Supplementary file 1 — Additional file 1. [file 12889_2020_8825_MOESM1_ESM.docx]

**In-Depth Interview Guide (Men)**

**Interviewer:** ______________________

**Notetaker (if applicable):** ______________________________

**Town/community/District:** ________________________

**Date:**_______________________

**Time discussion started:** __________  **Time ended:**_______________

**INTRODUCE INTERVIEWER, NOTE-TAKER:**

**INTRODUCE THE PURPOSE OF THE GROUP:**

Let’s go ahead and get started. My name is _____________________ and this is my colleague ____________________. We work for _____________________________. We are very interested in learning about VAW in this community. We will only share the information we learn today in a general way and in a way that does not reveal the identity of anyone. We really want to hear what you have to say and want you to feel comfortable in answering questions however you want to. There are no right or wrong answers.

________________ is taking notes to make sure that we don’t miss what you have to stay. This will help us later when we go back and organize all the information that was shared today. This interview should last for [insert amount of time].

Does anyone have any questions before we begin?

**Perceptions on Gender Relations**

***Women and men all over the world relate to each other. We are interested in knowing more about* how women relate with men generally, and in this community *[town name] specifically.***

1. In your opinion, in what ways can a man and woman be related to each other in this community/[name specific location]?

***IF INTIMATE RELATIONSHIP IS NOT MENTIONED🡪:*** What about getting intimately related to each other? Husband and wife? Boyfriend and girlfriend? Spouses/ Partners?

**We would like to learn more about the roles men and women play in this society as spouses or partners.**

3. What roles do women play in this community? What roles do men play?

**PROBE:** Domestic roles, occupational roles, leadership roles, others? Are women’s roles different from men’s roles? How? Are some roles rewarding than others? What makes one role more rewarding than the other? Are roles interchangeable?

**4.** How are decisions taken in households? Describe.

**PROBE:** Who takes the decision? Both spouses? One spouse? What decisions are usually taken by both spouses or by only one spouse? In what circumstances will decisions be taken by only one spouse? Describe. Why do you think some women might not be included in decision making by their partners/spouses?

5. How are misunderstandings between spouses/partners usually resolved?

**PROBE**: Between the spouses, or with a mediator? Is it done violently or amicably? Describe.

**AWARENESS, PERCEPTIONS, AND PRACTICES OF VAW**

**Now I’m going to ask you a few questions specifically about VAW (Violence Against Women).**

6. What kinds of VAW usually occur in this community? In what situations/circumstances does VAW occur?

**PROBE:** Describe. To what extent is VAW a problem in this /Local area?

**7.** Have you or someone close to you ever experienced VAW?

**PROBE:** Describe? When did this happen? How did this start? Why did it happen? Who was involved? How often do you experience this?

**8.** How will you describe the kind of violence you have just narrated?

**PROBE:** What about physical violence? Sexual Violence? Emotional Violence? Who is committing the violence? Boyfriends/Spouses? In what kinds of settings and circumstances did the violence occur? How often does it happen? What were the causes of the violence you experienced?

7. How will you describe the effects of VAW on you? On your family?

**PROBE:** How did you feel after experiencing the violence? Physically? Emotionally? Psychologically? Describe.

8. Personally how do you feel about the occurrence of VAW in this community?

**PROBE:** Is it something that you tolerate/allow? Why? How do you think VAW issues must be addressed in this community?

**SUPPORT FOR VAW VICTIMS**

9. When you experienced VAW what actions did you take? Did you go for support? Why? Why not?

**PROBE:** Where do women usually go for support if they experience violence? What about DOVVSU/Police? Health facility? CHRAJ? Family member? Neighbour? Social welfare? Probe for all sources and kinds of support given. Did you go to any of these institutions for support?

***PROBE:*** How did you find out about places that you can go for support?

**PROBE:** What kinds of support were given to you when you went? Were you satisfied with the support given to you? Why? Why not?

**Now imagine that you or a friend has just experienced VAW. Your friend goes for help. Afterwards when you see her, your friend tells you that getting that support was a really wonderful experience.**

10. What types of things do you think would make the support received a good experience for her?

**PROBE:** What kind of support will be given? What would they say? What would the setting be like?

11. What types of things would make getting support for VAW a bad experience for her?

**PROBE:** **PROBE:** What kind of support will be given? What would they say? What would the setting be like?

***Now think about an ideal situation in which anything is possible. In this ideal situation, there are plenty of resources and anything you want to happen can happen.***

12. In this ideal situation, how will men relate to women? How will VAW be addressed when it occurs?
